# Can Artificial Tree Cavities Provide Population Support to Cavity‐Dependent Non‐Flying Mammals?

**DOI:** 10.1002/ece3.74280

**Published:** 2026-09-04

**Authors:** Ross L. Goldingay, Karen J. Thomas, Darren G. Quin

**Affiliations:** ^1^ Faculty of Science and Engineering Southern Cross University Lismore New South Wales Australia; ^2^ Mandurang Victoria Australia

**Keywords:** Bendigo Regional Park, drought, habitat restoration, rainfall variation, tree hollow‐dependent mammals, tree hollows

## Abstract

Anthropogenic disturbances to forests have reduced densities of tree cavities and potentially led to declines in the abundance of cavity‐dependent mammals. Artificial cavities (nest boxes, carved and drilled cavities) may be used to restore habitat quality that may otherwise take many decades to recover naturally. We investigated whether artificial cavities could support populations of two species of Australian cavity‐dependent mammals, the brush‐tailed phascogale ( *Phascogale tapoatafa* , 200 g) and the inland sugar glider (*Petaurus notatus*, 110 g). This is the first detailed study of non‐flying mammals that has attempted to do this. We censused animals twice per year over 6 years in 164 nest boxes arranged in 74 clusters through a 600‐ha forest block deficient in tree cavities. We addressed four key questions: (1) were nest boxes used by a large number of individuals of the target species, (2) was use sustained over multiple years, (3) were nest boxes used for breeding and (4) what proportion of the local population might be supported by the nest boxes? We detected up to 58 adult phascogales and 232 adult sugar gliders in our boxes. Use was sustained over time with a mean probability of occupancy per cluster per year of 0.66 in the phascogale and 0.89 in the sugar glider. Differences in life history influenced how breeding was assessed. We detected breeding in 98% of female phascogales and at 68% of sites with sugar gliders. We estimated the total number of adults of each species that might occupy our study area based on their known spatial requirements. Our nest boxes potentially supported up to 61% of the adult female phascogales and 72% of the adult sugar gliders in the study area. Provision of shelter and breeding sites for such large segments of populations could be highly beneficial to manage populations of cavity‐dependent mammals.

## Graphical abstract

Artificial tree cavities may be used to restore habitat quality that may otherwise take many decades to recover naturally. We provide compelling evidence for two species of Australian cavity‐dependent mammals showing high and sustained usage of artificial cavities over 6 years, high levels of breeding within the cavities and support for large segments of the local population of each species.

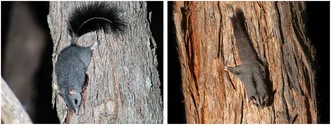

## 1 Introduction

Ecosystems worldwide are under continued threat from anthropogenic activities (e.g., Halpern et al. 2019; Finn et al. 2023). Forest ecosystems in particular have long been impacted by a multitude of threats, including widespread clearing for agriculture (Ward et al. 2019; Ke et al. 2023), urbanisation (Catterall et al. 1998), mining (Zhang et al. 2026), clear‐cutting and other forms of timber extraction (Green et al. 2022; Bousfield et al. 2024; Gorrod et al. 2024), clearing for roads and consequent fragmentation (Engert et al. 2024), an increasing frequency of wildfires (Abatzoglou and Williams 2016; Parks and Abatzoglou 2020; Collins et al. 2022), droughts (Dai 2013; Diffenbaugh et al. 2015; Cook et al. 2020) and more recently, climate change (Stouffer et al. 2021; Williams and de la Fuente 2021).

A widespread consequence of anthropogenic impacts on forests has been a decline in their age and structure (Jönsson et al. 2009; Lutz et al. 2009; Lindenmayer, Blanchard, et al. 2012). Tree cavities (or hollows) are key structural features that decline in abundance with reduced forest age (Newton 1994; Cockle et al. 2011; Zawadzka et al. 2016; Lindenmayer, Laurance, and Franklin 2012; Andersson et al. 2018; Ibarra et al. 2020; Altamirano et al. 2024; Gorrod et al. 2024). The decline in the availability of this resource is exacerbated by the long time‐horizons (> 100 years) for natural renewal (Mackowski 1984; Vesk et al. 2008; Manning et al. 2013). Many thousands of species of birds and mammals rely on tree cavities for shelter and/or breeding (Nowak 1999; Goldingay 2009, 2012; van der Hoek et al. 2017). Tree cavity excavator birds such as woodpeckers may create cavities used by many species, however, there are many regions worldwide where cavity users are dependent on cavities arising from fungal decay, insect attack and fire (Mackowski 1984; Bai et al. 2003; Wesołowski 2007; Cockle et al. 2011; Goldingay 2012; Bonaparte et al. 2024). The decline in the availability of such cavities forces species dependent on this resource into population declines if there is no management intervention (e.g., Manning et al. 2013; Lindenmayer et al. 2021; Davis 2022). Recommendations to protect existing large cavity‐bearing trees and potential recruitment trees are worthwhile but likely insufficient to prevent the decline in abundance of cavity‐dependent species due to the overall shortage of such trees and the time lags for hollow development.

The principal form of management intervention to increase the availability of tree cavities is to install artificial cavities. These have most commonly been in the form of nest boxes, but chainsaw‐carved cavities and drilled cavities have become more common in recent years (Carey 2002; Katzner et al. 2005; Brazill‐Boast et al. 2013; Goldingay 2025; Best et al. 2026). Whilst many studies have involved the installation of artificial cavities, study objectives have been varied and not necessarily directed at the support of populations of cavity dependent birds and mammals (Beyer and Goldingay 2006; Goldingay 2025). Many studies have used artificial cavities to facilitate investigation of various attributes of bird breeding (e.g., Lambrechts et al. 2010). One topic of direct relevance to the effectiveness of artificial cavities in supporting populations is whether there are fitness consequences for animals using nest boxes, with most studies showing a positive or a neutral effect (e.g., Purcell et al. 1997; Fargallo et al. 2001; Brazill‐Boast et al. 2013; Singh et al. 2016; Goldingay 2017).

There is evidence from Europe (Libois et al. 2012; Habel et al. 2015; Kiss and Tokody 2017; Bank et al. 2019; Fay et al. 2019), North America (Carle‐Pruneau et al. 2022), Britain (Petty et al. 1994), Micronesia (Savidge et al. 2022), New Zealand (Briskie et al. 2014) and Australia (Brazill‐Boast et al. 2013; Saunders et al. 2020; Berris et al. 2018; Berris et al. 2022) that nest boxes can support populations of cavity‐dependent birds. Whilst there is some evidence that artificial cavities provide population support for some cavity‐using bats (e.g., Flaquer et al. 2006; Vergari and Dondini 2011; Griffiths et al. 2018; Printz et al. 2021), there are some ethical concerns associated with this management practice due to risks associated with overheating (Martin Bideguren et al. 2018; Griffiths 2022) and possible disruption of local species composition by some species that readily use bat boxes (Matus‐Olivares et al. 2025; but see Griffiths, Lumsden, et al. 2020). Evidence for non‐flying mammals consists of just two cases in Australia where a species of gliding mammal was supported by nest boxes when translocated to isolated forest patches, with the resulting populations persisting for at least 30–45 years (Goldingay 2025).

Despite this growing evidence that artificial cavities can support populations of cavity‐dependent species, there are some concerns around whether such a management intervention can be successful (see Lindenmayer et al. 2009). These relate to whether the target species are the main beneficiaries of artificial cavities, and whether the artificial cavities can be maintained and their use sustained over long periods of time. The studies cited earlier demonstrate successful targeting of threatened or declining species. Other studies provide evidence that artificial cavities remain useful to target species for periods of > 20 years (Schölin and Källander 2011; Goodwin et al. 2017; Griffiths et al. 2018; Quin et al. 2021) but more studies are required.

The question of whether artificial cavities can be demonstrated to support a population could be approached in different ways. The most compelling would be to conduct an experimental study with a before‐after‐control‐impact design (e.g., Aitken and Martin 2012; Lima and Garcia 2016; Griffiths, Semmens, et al. 2020; Altamirano et al. 2024) but at a landscape scale rather than at a plot or stand scale. Such an approach is challenging due to the cost and logistics involved when attempting to replicate across landscape units. An alternative approach is to demonstrate at a landscape scale that a large segment of a population has used artificial nest sites for breeding and is continuing to be supported (for shelter and/or breeding) by the artificial nest sites. This approach is particularly relevant where only a single occupied landscape is available for study and may have already been subject to management interventions to prevent population decline (e.g., Arlettaz et al. 2010; Saunders et al. 2020; Berris et al. 2022).

**FIGURE 1:**
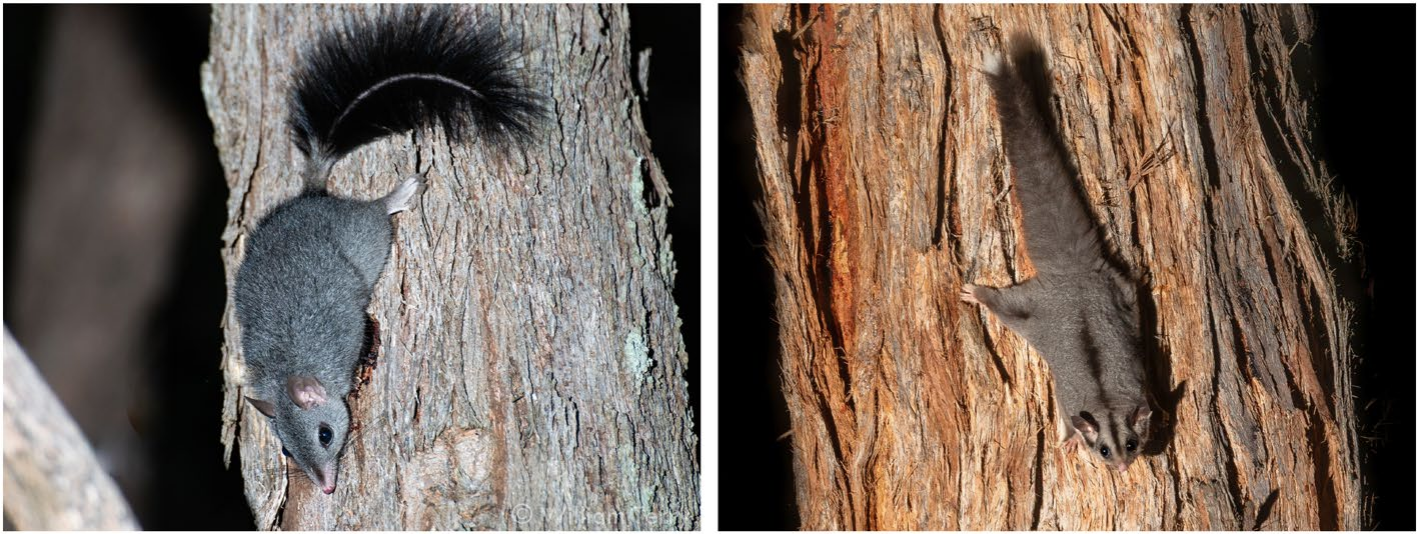
Brush‐tailed phascogale (*left*). Inland sugar glider (*right*). Images: William Terry.

We investigated whether nest boxes installed in a degraded 600 ha forest patch provide population support to two species of tree cavity‐dependent mammals: the brush‐tailed phascogale ( *Phascogale tapoatafa* ; 200 g) and the inland sugar glider (*Petaurus notatus*; 110 g) (Figure 1). Both rely on tree cavities for shelter and in which to raise young (Suckling 1984; Traill and Lill 1997; van der Ree et al. 2006; Terry and Goldingay 2026). Our study forest had been subject to broad‐scale historic clearing and timber extraction and contained a low abundance (0–1.3/ha) of trees with cavities suitable to either species for breeding (Goldingay et al. 2024). Equivalent unlogged forest may contain as many as 20 such cavity‐bearing trees per ha (van der Ree et al. 2001).

To demonstrate that artificial cavities can support (i.e., provide for) a local population requires four separate types of evidence: (1) that a large number of individuals of a target species are using the cavities, (2) that the level of use is sustained over multiple years, (3) that many if not most of the individuals use the cavities for breeding and (4) that the number of individuals using the cavities represents a sizeable segment (e.g., > 25%) of a local population. We conducted biannual surveys of nest boxes over 6 years to test points one and two. Nest box use might wax and wane for various reasons, such as a drought that occurred in year two of our study, so periodic surveys over 6 years would reveal whether a non‐trivial percentage of sites was consistently occupied. Two surveys within a year allowed us to evaluate whether our target species were breeding in the nest boxes (point 3). One was timed later in the year to coincide with the nestling phase of the phascogale (Soderquist 1993a; Goldingay, Quin, et al. 2020) and the sugar glider (Suckling 1984). Both species have relatively extended periods (2–4 months) of nestling development such that breeding would not be overlooked. The fourth point was evaluated by estimating the total number of individuals that the forest area could support if not limited by tree cavity abundance (by assuming densities equivalent to what has been estimated in equivalent habitat) and comparing to the number we observed in nest boxes. Most previous studies of non‐flying mammals and nest boxes have used nest boxes as a census technique (Goodwin et al. 2017), or to investigate habitat selection (Williams et al. 2013; Mortensen et al. 2022) or nest box design preferences (Delheimer et al. 2018; Goldingay 2025), which are important first steps before attempting population management. Our focus was on evaluating the value of nest boxes for population management.

## 2 Methods

### 2.1 Study Area

**FIGURE 2:**
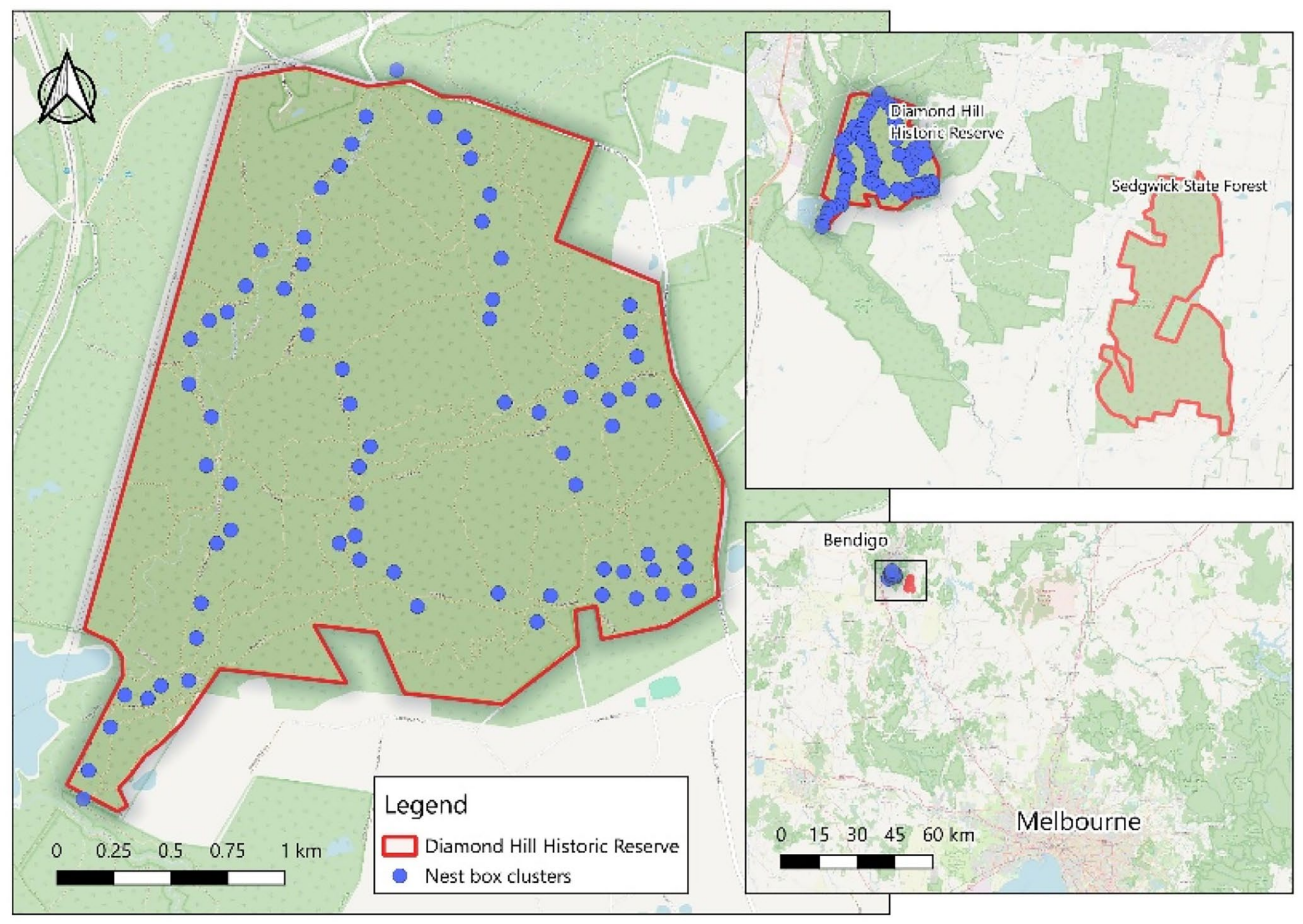
The study area near Bendigo, north‐west of Melbourne, Victoria, Australia and the location of the 74 nest box clusters. The upper right inset shows the location of Diamond Hill (left) and Sedgwick State Forest (right).

This study was conducted at Diamond Hill (36°50′10″ S, 144°15′48″ E), in a section of Bendigo Regional Park near Bendigo, Victoria, Australia (Figure 2). The forest of our study area, which covered 600 ha, was not isolated from other expanses of forest but was surrounded by 15‐m wide arterial roads to the east and north, low density residential development to the south, a large reservoir to the south‐west and urban development bordered by a major railway line and an 80‐m wide powerline easement to the west. The forest contained the following plant communities: *box‐ironbark forest, heathy dry forest*, *alluvial terraces herb‐rich woodland* and *creekline grassy woodland*. Historic clear‐felling of the forest associated with gold mining had produced a forest dominated by coppice regrowth approximately 80 years old (Goldingay et al. 2018). Elevation ranged from 275 to 360 m. Annual rainfall at Eppalock Reservoir (20 km away) averaged 509 mm and varied during our study (2018–2023) from 46% of average in 2019 and 52% in 2020, to 151% in 2022 (Bureau of Meteorology, bom.gov.au). At Bendigo airport mean maximum monthly temperature in the warmest month was 30.3°C (range 26.3°C–35.6°C) and mean minimum monthly temperature in the coldest month was 2.7°C (range 0.4°C–5.4°C) (bom.gov.au).

### 2.2 Artificial Hollows

Nest boxes were the artificial hollows used in this study. Community‐led projects had installed > 100 nest boxes along 4WD trails across the study area > 20 years prior to our study and were being used by our focal species prior to the current study (see Goldingay et al. 2018). These were repaired and augmented in 2017, so when our study commenced in 2018 there were 142 functional nest boxes and had been in place for 1 year. Further augmentation brought the total to 164. The boxes were of various designs due to the history of installation and construction by different individuals. One‐third (0.33) were made of PVC tubing while the remainder were made of ply (0.18), hardwood (0.25) or pine (0.25). The different designs appeared not to influence use (Goldingay et al. 2024). All boxes showed evidence of use based on the presence of nests of either focal species over the 6 years. The PVC boxes were mostly 25‐cm in diameter, 30–35 cm in height and capped top and bottom by sheet metal and lined with a 1‐cm thick wood composite. Timber boxes measured 20 cm (wide) × 10–20 cm (deep) × 20–40 cm (high). Boxes had entrance holes of 3.5–4.5 cm diameter. They were hung on a single large nail at a height of 3–4 m and positioned on the east side of trees to minimise solar radiation in the afternoon. The nest boxes were arranged in 74 clusters (i.e., sites), that were spaced 100–300 m apart. A cluster contained 1–4 boxes spaced 5–50 m apart. The spacing was partly a consequence of the history of nest box installation in the study area. The clusters were distributed along the major management trails giving broad coverage through the forest (Figure 2). We recognise the clusters as independent sites which is fundamental to our analyses described below. Both species change nest sites frequently and have home range sizes that readily encompass a cluster (Quin et al. 1992; Soderquist 1995). The brush‐tailed phascogale may use 3–7 dens per 10 observation days (van der Ree et al. 2006; Terry and Goldingay 2026) whereas the squirrel glider ( *P. norfolcensis* ), a congener of the inland sugar glider, may change dens every 3–5 days (Crane et al. 2010).

### 2.3 Animal Surveys—How Many Individuals Used the Nest Boxes?

We timed our nest box inspections to encompass different periods of the reproductive cycles of the brush‐tailed phascogale and the inland sugar glider. Nest boxes were inspected twice per year (usually May and October) for 6 years from 2018 to 2023. The phascogale has a semelparous life history with complete male die‐off occurring in June in Victoria (Cuttle 1982). Consequently, only adult female phascogales with young are present in October. We chose not to tag any animals, so our inspections consisted mostly of counting individuals within the boxes from a 3 m long ladder. We hand‐captured phascogales and sexed them from 2019 onwards to clarify the number of adult males present in May. Our first survey in 2021 was delayed until late June by which time only females were present and 19% had small pouch young. Our inspections occurred over a 2‐day period and followed a spatial sequence that meant no double counting of individuals could occur.

### 2.4 Multi‐Year Dynamics of Nest Box Occupancy—Was the Level of Use Sustained Over Multiple Years?

Previous studies of non‐flying mammals using nest boxes for > 3 years provide little guidance to know whether levels of use can be sustained over time. Mostly, formal analysis was not conducted and/or levels of use were low (Carey 2002; Harley 2004; Lindenmayer et al. 2009; Williams et al. 2013; Quin et al. 2021; Lawton et al. 2022). In others, use showed wide fluctuation (Shuttleworth and Schuchert 2014), or the focal species (hazel dormice *Muscardinus avellanarius* ) was in long‐term decline (Goodwin et al. 2017; Scopes et al. 2023). Therefore, a rigorous approach was required to conduct our evaluation.

We used multi‐season occupancy modelling (MacKenzie et al. 2003) to describe the multi‐year dynamics of nest box use. This modelling approach estimates initial site occupancy (psi, ψ) from which annual fluctuations due to site colonisation (gamma, ɣ) and local extinction (eps, ɛ) can be estimated. The extinction parameter was used to estimate persistence at a site (phi, Φ = 1 − ɛ) (MacKenzie et al. 2018). This parameter has not been used previously in studies of nest boxes to index stability of use of a site over time. It can be viewed as a measure of the ongoing value of the site when artificial cavities are provided. This is best reflected by the phascogale which has a short lifespan (males: < 11 months; females: 77% < 18 months; Cuttle 1982; Soderquist 1993b) so mostly different individuals were evaluating the nest box sites anew each year. The occupancy model also estimates detection (p), which is important for estimating the other parameters because some animals potentially had access to natural tree hollows such that they may avoid nest boxes or use them intermittently (e.g., Goldingay 2017; Terry and Goldingay 2026). Furthermore, the population dynamics relating to site (box) colonisation and extinction are not simply a reflection of acceptance of the suitability of the boxes but may be influenced by extrinsic factors such as variation in rainfall that will induce variation in abundance and therefore detection (e.g., Goldingay et al. 2025).

We conducted analyses in program presence version 15.09 (USGS Patuxent Wildlife Research Centre, Laurel, MD, 20708, USA). We constructed detection histories for each site (i.e., cluster) to indicate during each survey occasion whether each species was detected (1) or not (0) or a site was not established (−). An assumption of occupancy analysis is that sites have independent detection histories (MacKenzie et al. 2003) thus precluding treating individual boxes as sites. We analysed the phascogale data in two different ways to account for the annual male die‐off between our two annual surveys. First, we constructed detection histories using detection of any phascogale. Second, we constructed detection histories using only the female detections. We excluded data for 2018 because we did not sex phascogales in our first survey.

We fitted models to be year‐constant or year‐varying. We used the number of nest boxes as a site covariate which was centred by subtracting the mean and dividing by the standard deviation. We used the method of MacKenzie and Bailey (2004) to assess model fit using the most general single‐season occupancy model. This was implemented in presence using 10,000 bootstrap samples. The test statistic suggested there was an adequate fit to the data for all phascogale data (*p* = 0.45) or for the female only data (*p* = 0.47). However, there was evidence of a lack of fit in the sugar glider data (*p* = 0.001; $\hat{c}$ = 4.4). The value of ĉ was used to adjust for overdispersion which led to comparison of models using quasi‐AICc (QAICc) and the standard errors adjusted by the $\surd \hat{c}$ (MacKenzie and Bailey 2004).

We compared models using the Akaike Information Criterion corrected for small sample size (AICc) (Burnham and Anderson 2004). Models were ranked by presence from lowest to highest AICc, with higher ranked models having more support in explaining the data. Differences in AICc (∆AICc) between the top model and other models indicate the level of support for those competing models (Burnham and Anderson 2004). Models are equally plausible if ∆AICc is < 2. Models have less support as this value increases.

The main factor of interest in our modelling was whether year‐varying models had more support than year‐constant models. We recognise that models that allow every year to be estimated as different may add unnecessary complexity (see Arnold 2010) so we also fitted models that allowed some years to be different and some equivalent. The parameter estimates from the year‐varying model were used to guide construction of the some‐years‐different model. We modelled detection with the site covariate included for initial occupancy. We then modelled colonisation and extinction before again modelling occupancy to ensure the sequence has not influenced model selection. Models that did not converge were excluded.

### 2.5 Were Nest Boxes Used for Breeding?

Both species raise young in October and early November (Smith 1979; Cuttle 1982; Suckling 1984; Soderquist 1993a, 1993b). Solitary female phascogales raise a litter in a maternal den that consists of a large, enclosed chamber within a bark nest (Soderquist 1993a; Goldingay, Quin, et al. 2020; Goldingay et al. 2024). Inspection at that time often required placing a gloved hand into the chamber to detect if nestlings were present. If not, the adult female was hand‐captured and her pouch examined for young. In contrast, sugar gliders live in small family groups throughout the year comprised of multiple adults and subadults (Suckling 1984). We made an approximate count of the number of adults, subadults and nestlings present in a group. Subadults and nestlings are noticeably smaller than adults. Their presence indicated that a breeding group was present at a site. Due to some ambiguity with different observers in 2022 and 2023, only the breeding counts made by RLG were used. None were included for October 2021 due to Covid19 travel restrictions in Victoria that prevented RLG and DGQ from participating in the survey.

### 2.6 What Proportion of a Local Population Was Supported by the Nest Boxes?

Our intention here is to estimate the level of population support (i.e., the proportion of the local population) that our nest boxes provided. We estimate both a theoretical population size if the Diamond Hill populations were not limited by the abundance of tree cavities and a more realistic size by accounting for the reduced abundance of cavities in the forest. We assume that animals using our nest boxes were dependent on them and not on any natural cavities.

The following assumptions were made to estimate the theoretical population sizes: (1) that the mean multi‐year occupancy of each species indicated how much of the forest habitat through Diamond Hill was suitable to each species, and (2) the density of each species at Diamond Hill was equivalent to that described in similar habitat at other locations and could be multiplied by the area of suitable habitat. Our population estimates for the phascogale are for adult females only. Adult female brush‐tailed phascogales are territorial whereas males are not (Soderquist 1995), which means the area of the territory can be used to estimate the number of adult females that could be supported across the forest and therefore provide a more reliable index of the population than if males were included. Females in southern Australia occupy forest areas of 25–78 ha. Smaller core areas of 4–13 ha are used intensively over periods of 1–3 months and show minimal overlap among neighbouring individuals (Soderquist 1995; van der Ree et al. 2001). The mean core area of three populations was estimated to be 10 ha (i.e., a density of 0.1 per ha) (Soderquist 1995).

Choosing an appropriate density value for the inland sugar glider was less straightforward. Densities of 2–3.6 inland sugar gliders per ha have been estimated for fragmented road‐side habitat in Victoria (Suckling 1984). However, a primary food item in that habitat was the gum of black wattle ( *Acacia mearnsii* ) (A. Smith 1982) and glider density was positively associated with its abundance (Suckling 1984). This plant and *E. bridgesiana* , which provided another important food of tree sap, were both absent from our study area and there was no evidence that gum or sap were derived from other plants, so equivalent glider densities are very unlikely in our study area. In a drier tablelands forest in northern NSW a density of 0.29–0.63 individuals per ha can be estimated from a 35‐ha trapping grid with a 50‐m buffer (Smith and Phillips 1984). Kavanagh (1984) estimated densities of just 0.1 per ha on the NSW southern tablelands. Braithwaite (1983) estimated densities of < 0.1 per ha based on clear‐fell logging through a broader area of south‐east NSW at a mean elevation of < 300 m. Density of the coastal sugar glider ( *Petaurus breviceps* ) has been estimated at 0.1–0.7 individuals per ha (Quin 1995; Gracanin et al. 2022). We use an estimate of 0.5 per ha to represent the number of adults. This is in the upper range of the above values which include adults and subadults.

The more realistic population estimates assume the populations of the two species consisted of two segments, one relying on the nest boxes and another away from the nest boxes which we assume were at half the density. This latter assumption is plausible given the documented scarcity of cavities suitable for breeding in the study area (Goldingay et al. 2024). In forest where the number of tree cavities varied substantially, the number of Leadbeater's possums ( *Gymnobelideus leadbeateri* ) (Smith and Lindenmayer 1988), mountain brushtail possums ( *Trichosurus cunninghami* ) and greater gliders ( *Petauroides volans* ) (Lindenmayer, Cunningham, et al. 1990) declined by > 50% across a gradient of cavity availability from abundant to scarce. In habitat with a dearth of natural cavities suitable for breeding the number of adult eastern pygmy‐possums on plots provisioned with breeding cavities was 1.5–3 times higher than on plots not provisioned (Goldingay 2020a). In forest in which the number of cavity‐bearing trees declined substantially over 20 years, declines in occurrence of at least 50% occurred in the inland sugar glider and two other species of cavity dependent non‐flying mammal (Lindenmayer et al. 2021).

We used the October censuses to calculate the annual proportions of the population sizes relying on the nest boxes. Only adult female phascogales were present at that time. The adult sugar glider counts were higher in October and perhaps more indicative of the local population. The counts were divided by the theoretical population sizes to show the proportion in the boxes each year. The theoretical size was a fixed value each year despite observed interannual variation in animal counts which appeared to be driven by rainfall variation. We used the mean of the annual counts in our more realistic population size estimates. To estimate the size of the population segments that were not supported by the nest boxes we simply subtracted the 6‐year mean count from the theoretical population sizes. We then reduced these values by 50%. This value plus the annual count equalled the population size under this scenario. The population size estimates assume the abundance of one species does not influence the other species. We found no evidence of detection of one species influencing the detection of the other across 40 of the nest box sites in our study area (Goldingay et al. 2024). Any interaction in areas away from the nest box sites might reduce the size of those population segments, suggesting the nest boxes might support higher proportions of the overall populations.

### 2.7 Camera Trapping to Index Animal Activity Near and Far From Nest Boxes

We conducted camera‐trapping to investigate whether the abundance of our target species was influenced by the nest boxes. This was conducted between January 2023 and February 2024. We predicted that detection of phascogales and sugar gliders near occupied nest box sites would be higher than at sites without nest boxes. Whilst detection may reflect animal activity it can also be influenced by animal abundance (Royle and Nichols 2003). We established 10 replicate sites of three different types for monitoring: sites ‘near’ a previously occupied nest box, sites at least 300 m from a nest box site (i.e., ‘far’) and sites within another forest area (Sedgwick State Forest, Figure 2) without any nest boxes (i.e., ‘no nest box’). The 10 sites ‘near’ a nest box were randomly chosen from 29 used as maternal nests by phascogales in 2022 and selected to provide broad coverage through the study area. All but two were located at least 500 m apart. Four of these sites were not used for breeding by phascogales in 2023 and a different nest box to that used in 2022 was used for breeding at 3 sites. Two sites were not used by sugar gliders in 2023. A camera was installed on a tree within a 10 m radius of the target nest box.

The 10 sites ‘far’ from a nest box site were in a randomly chosen direction. The spacing of 300 m was chosen to place the survey site beyond the 4–13 ha area (radius of ~200 m) used intensively by a breeding female phascogale (Soderquist 1995). Sedgwick Forest (approximately 1300 ha in area) is located 4.6 km southeast of Diamond Hill and contained similar forest types. Tree cavities suitable for breeding by our study species were equally uncommon through this forest. Sites were randomly located along management trails throughout the forest block. To ensure our monitoring of sites without nest boxes (i.e., ‘far’ and ‘no nest boxes’) was not biased by a complete absence of tree cavities we searched to locate a tree with a cavity of sufficient size for a phascogale (a single entrance of 2.5–6 cm diameter; at least 10 cm deep) at each of the ‘far’ and Sedgwick (‘no nest box’) sites. Replicate sites were at least 500 m from each other. Cameras were installed on a tree within a 10 m radius of these trees. Two cameras in Sedgwick were repositioned 50 m away after the initial camera was stolen. Cameras were mounted on metal stakes and directed at a tree 2–3 m away on which a vented canister (PVC tube, 5 × 15 cm) containing bait (peanut butter, oats, maple syrup and fish oil) was installed at 1.5 m high. This was a combination bait to be attractive to both the phascogale (Lawton et al. 2021) and sugar glider (Owens et al. 2024). Cameras were serviced (SD cards replaced; bait renewed) approximately every 2 months. Cameras (Swift Enduro and Swift 3C wide‐angle cameras) were set to take 5 images when triggered with a 1 min quiet period between triggers.

The camera‐trapping data were analysed using single season occupancy modelling (MacKenzie et al. 2002). This modelling estimates the probability of detection and the probability of occupancy. Our study species were detected at all but one survey site so the probability of occupancy was fixed to 1.0. Our focus was on whether our study species were detected equally at the three types of camera sites described above. We constructed detection histories for each site to indicate whether each species was detected (1) or not (0) or a camera was not present or functioning (−) during each survey occasion. We conducted analyses in program presence version 15.09 (USGS Patuxent Wildlife Research Centre, Laurel, MD, 20708, USA).

We compared five different detection models: (1) a null model in which detection was constant across all occasions and sites; (2) a season‐varying model (i.e., each season different); (3) a model where only some seasons were different; (4) a model in which detection was influenced by site location (near nest box, far from nest box, no nest box); and (5) a model in which detection differed for sites near a nest box compared to both types without a nest box. The latter two models are consistent with the advice of Arnold (2010) to not include uninformative parameters. We predicted that season would be influential and particularly on the phascogale given male die‐off in winter and that young do not leave the nest until summer (Soderquist and Lill 1995; Rhind and Bradley 2002). We used AICc as described above.

## 3 Results

### 3.1 General Observations

Over the 6 years the lowest number of phascogales (both sexes) recorded in the boxes was 20 in May 2020. The highest number was in May 2019 when 58 were recorded. Naïve occupancy ranged from 0.31 in 2020 to 0.58 in 2023. The lowest number of sugar gliders was 70 adults in October 2019 in the main drought year, and the highest number was 316 individuals (220 adults, 66 subadults and 30 nestlings) in October 2023, occupying 67 boxes, following 3 years of above‐average rainfall. Naïve occupancy ranged from 0.70 in 2019 to 0.89 in 2022.

### 3.2 Were the Cavities Used by a Large Number of Individuals of the Target Species, and Was Use Sustained Over Multiple Years?

#### 3.2.1 All Brush‐tailed Phascogales

**FIGURE 3:**
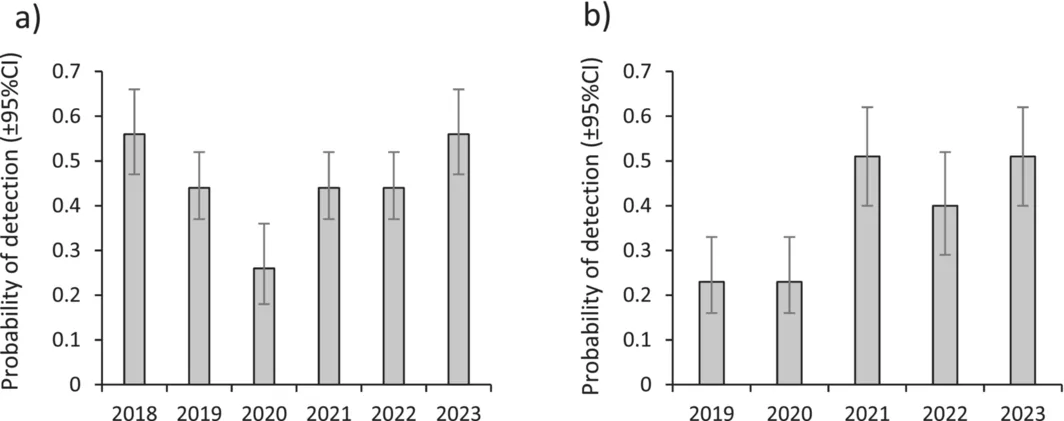
Annual estimates of the probability of detection for (a) all phascogales (without the nest box covariate) and (b) female phascogales only.

**FIGURE 4:**
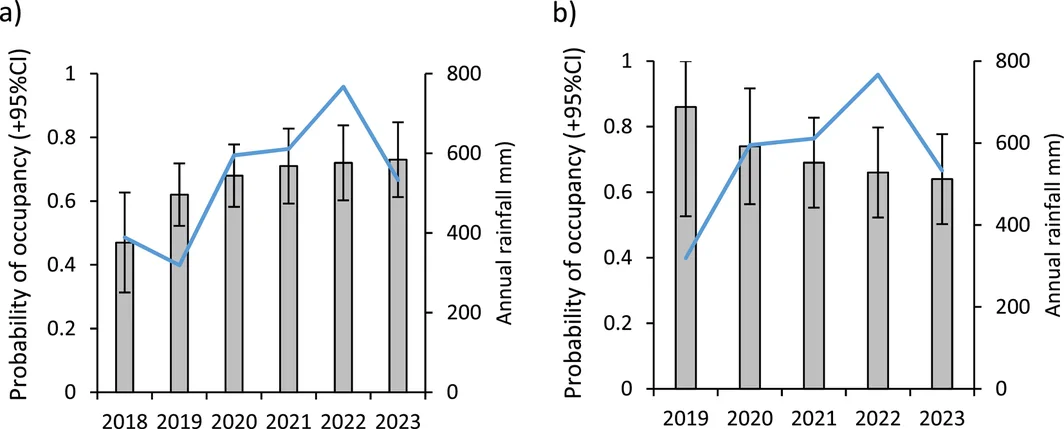
Estimates of the probability of occupancy for (a) all phascogales (with nest boxes set at 2) and (b) female phascogales only. The blue line shows annual rainfall. The long‐term average is 509 mm.

The most plausible model allowed detection to be equivalent in some but not all years and included the number of nest boxes as an influence on occupancy (Table 1). Allowing some years to differ had much more support than allowing all years to differ. The probability of detection was much lower in 2020, the year after the main drought year, compared to all other years (Figure 3a). The number of nest boxes had a positive influence on initial occupancy (β = 0.94 ± 0.47 (SE)). The top model allowed for the probability of colonisation and extinction to be constant across year periods and was estimated at 0.41 ± 0.07 and 0.15 ± 0.05, respectively. The probability of persistence was 0.85 ± 0.05. The probability of occupancy showed a slight increase over time, but the confidence intervals overlapped (Figure 4a). Over the 6 years it averaged 0.66.

**TABLE 1:** Comparison of occupancy models for the brush‐tailed phascogale.

Model	AICc	∆AICc	*W*	*k*
All phascogales				
Psi(boxes), gamma(.), eps(.), p(some years)	1028.38	0.00	0.88	7
Psi(.), gamma(.), eps(.), p(some years)	1033.35	4.97	0.07	6
Psi(boxes), gamma(.), eps(.), p(years)	1035.66	7.28	0.02	10
Psi(boxes), gamma(years), eps(.), p(some years)	1036.79	8.41	0.01	11
Psi(boxes), gamma(.), eps(years), p(some years)	1037.60	9.22	0.01	11
Psi(.), gamma(.), eps(.), p(.)	1046.79	18.41	0.00	4
Females only				
Psi(.), gamma(.), eps(.), p(some years + boxes)	801.64	0.00	1.00	7
Psi(.), gamma(.), eps(.), p(boxes)	816.75	15.11	0.00	5
Psi(.), gamma(.), eps(.), p(some years)	820.06	18.42	0.00	6
Psi(.), gamma(.), eps(.), p(.)	832.33	30.69	0.00	4

*Note:* Models include parameters for occupancy (psi), colonisation (gamma), local extinction (eps) and detection (p). *W*, model weight; *k*, number of parameters. Covariates: (.), null; some years, some years different; years, all years different; boxes, number of nest boxes per site.

#### 3.2.2 Female Brush‐tailed Phascogales

The most plausible model allowed detection to be equivalent in some but not all years (Table 1). The probability of detection was lowest in the main drought year of 2019, and the year after (Figure 3b). Models that included the number of nest boxes did not converge. The top model allowed for the probability of colonisation and extinction to be constant across year periods and were estimated at 0.32 ± 0.11 and 0.19 ± 0.07, respectively. The probability of persistence was 0.89 ± 0.06. The probability of occupancy showed a slight decrease over 5 years (average 0.72), but the confidence intervals overlapped (Figure 4b).

#### 3.2.3 Sugar Gliders

**FIGURE 5:**
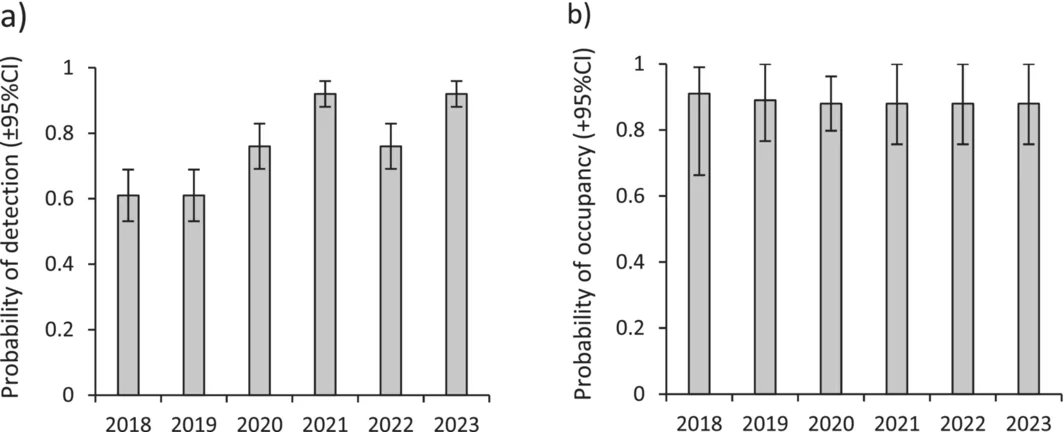
Estimates of the probability of (a) detection and (b) occupancy in the sugar glider.

The most plausible model allowed detection to be equivalent in some years but to differ in others (Table 2). This model had much more support than allowing detection in all years to differ. The probability of detection was estimated to be lowest in 2018 and 2019 compared to all others (Figure 5a). Models that included the number of nest boxes did not converge. The probability of occupancy was estimated at > 0.80 (average 0.89) throughout the 6 years (Figure 5b). The probability of colonisation and extinction was constant across periods and estimated at 0.54 ± 0.21 and 0.08 ± 0.04, respectively. The probability of persistence was 0.92 ± 0.04.

**TABLE 2:** Comparison of occupancy models for the sugar glider.

Model	QAICc	∆QAICc	*W*	*k*
Psi(.), gamma(.), eps(.), p(some years)	236.83	0.00	0.95	6
Psi(.), gamma(.), eps(.), p(years)	244.02	7.19	0.03	9
Psi(.), gamma(.), eps(.), p(.)	244.25	7.42	0.02	4

*Note:* Model parameters as for Table 1.

### 3.3 Were Nest Boxes Used for Breeding?

**FIGURE 6:**
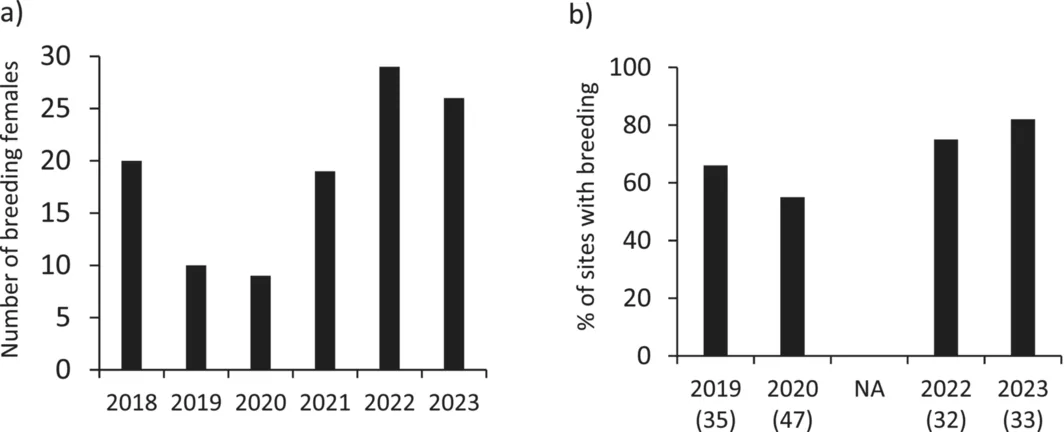
Frequency of breeding over the study period. (a) brush‐tailed phascogale and (b) sugar glider. Values below the year for the sugar glider show the sample size of sites. Data were not collected in 2018 and 2021.

**FIGURE 7:**
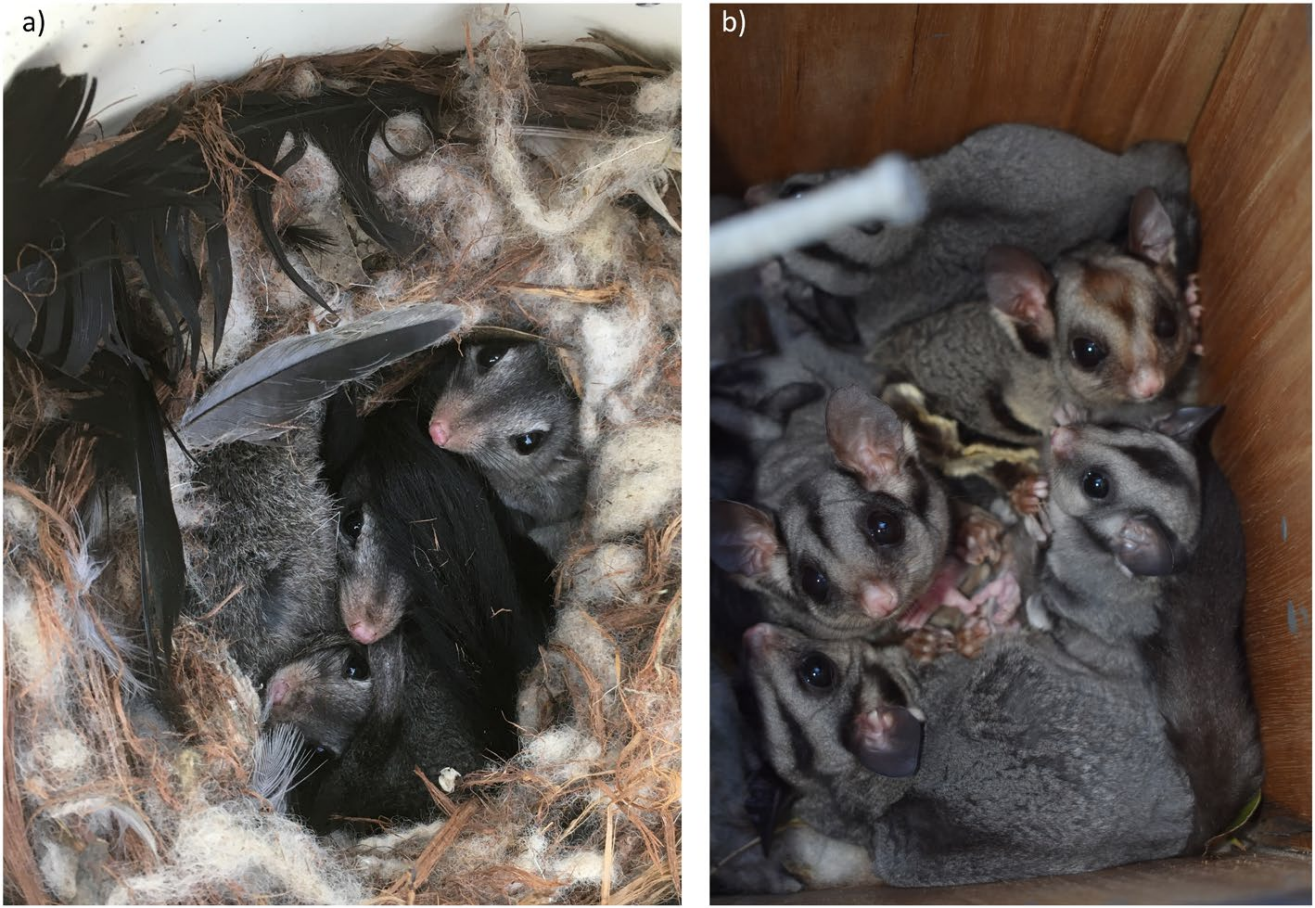
(a) A phascogale maternal nest with two nestlings visible. (b) A sugar glider group with smaller bodied subadults among adults. Images: R. Goldingay.

The number of female phascogales with young varied from 9 in 2020 to 29 in 2022 (Figure 6a). We did not attempt to record the number of nestlings that were present (Figure 7a) to minimise disturbance; however, in many cases there were at least 4 young. This is consistent with the findings for other populations (Soderquist 1993a; Rhind 2002). In 2018 we observed one box with 3 dead young but no adult. In 2019 we observed an adult female phascogale in each of two boxes with no young and one box, distant to the other two, where there were 7 dead young but no adult. Overall, 98% of 116 adult females detected within the nest boxes in the breeding season were observed to have young. In the sugar glider we relied on observing the presence of subadults or nestlings to indicate breeding (Figure 7b). We documented some instances where adult females had pouch young in the absence of subadults or nestlings. Therefore, our approach was conservative but revealed that glider groups at 68% of 147 sites (range: 55%–82%) showed evidence of breeding (Figure 6b).

### 3.4 What Proportion of the Local Population Might Be Supported by the Nest Boxes?

The theoretical population sizes were estimated to be 43 female phascogales (600 ha × 0.72 occupancy × 0.1/ha) and 267 adult sugar gliders (600 × 0.89 × 0.5). The number of individuals in our nest boxes varied over the study period, representing a mean of 0.44 of the theoretical population size (range 0.21–0.67) of a local population of adult female phascogales and a mean of 0.56 (range 0.26–0.87) of a local population of adult sugar gliders if we assume density is at 100% throughout the forest in a scenario where there is no shortage of breeding cavities (Table 3). However, if density is at 50% away from the nest boxes due to a shortage of breeding cavities, we estimate our cavities supported 0.61 (range 0.29–0.94) of 31 adult female phascogales and 0.72 (range 0.33–1.11) of 209 adult sugar gliders.

**TABLE 3:** The number of adult sugar gliders and adult female phascogales recorded in nest boxes (NB) across years (Count) and proportion of the population supported under two scenarios.

Year	Phascogales			Gliders		
	Count	100%	NB + 50%	Count	100%	NB + 50%
2018	20	0.47	0.65	85	0.32	0.41
2019	11	0.26	0.35	70	0.26	0.33
2020	9	0.21	0.29	131	0.49	0.63
2021	19	0.44	0.61	232	0.87	1.11
2022	29	0.67	0.94	164	0.61	0.78
2023	26	0.60	0.84	220	0.82	1.05
Mean	19	0.44	0.61	150	0.56	0.72

*Note:* These are (i) the theoretical local population if tree cavities were not limiting (100%) when we estimate there are 43 adult female phascogales and 267 adult sugar gliders; and (ii) a more realistic estimate if density is at 50% in areas without nest boxes because tree cavities are scarce when we estimate there are 31 adult female phascogales and 209 adult sugar gliders.

### 3.5 Camera Trapping to Index Animal Activity Near and Far From Nest Boxes

**FIGURE 8:**
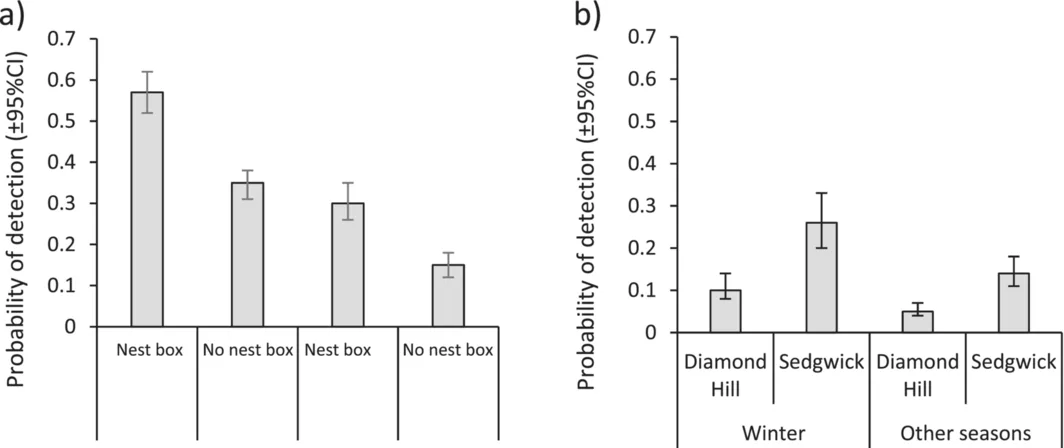
Estimates of the probability of detection from camera trapping in (a) the brush‐tailed phascogale and (b) the sugar glider. Differences are shown between two season groups. In the phascogale there is also a contrast between sampling near a nest box cluster and where there was no nest box nearby. In the sugar glider there is also a contrast between the two forest areas.

The detection model for the brush‐tailed phascogale with overwhelming support was one that included a contrast in two season groups and a contrast in the camera locations (Table 4). Models in which all four seasons and three locations were estimated as different showed much less support and are not shown. There was an influence of camera type, but its influence was much less than that of season or camera location. Detection probability showed a marked difference between seasons, being approximately twice as high in summer and autumn compared to winter and spring (Figure 8a). There was also a strong influence due to the camera location. Cameras located near a nest box cluster had detection probabilities on average 1.7 times higher than cameras located where there was no nest box nearby. This supports our hypothesis that phascogale abundances are influenced by the nest boxes and that phascogale densities are likely to be substantially lower where no nest boxes are installed.

**TABLE 4:** Comparison of detection models from camera‐trapping for the brush‐tailed phascogale.

Model	AICc	∆AICc	*W*	*K*
p(2 seasons+2 locations)	1798.84	0.00	1.00	4
p(2 seasons+camera)	1852.98	54.14	0.0	4
p(2 seasons)	1857.51	58.67	0.0	3
p(2 locations)	1889.55	90.71	0.0	3
p(camera)	1939.14	140.30	0.0	3
p(.)	1943.19	144.35	0.0	2

*Note:* Model parameters as for Table 1. Covariates: 2 seasons, summer/autumn contrasted with winter/spring; 2 locations; contrast of nest box and no nest box sites; camera, contrast of 2 camera models.

The detection model for the sugar glider with overwhelming support was one that included a contrast in two season groups and a contrast in the camera locations (Table 5). This had much more support than the null detection model. Models in which all four seasons and three locations were estimated as different had much less support. Camera type had no influence. Detection probability showed a marked difference between seasons, being approximately twice as high in winter compared to other seasons (Figure 8b). There was also a strong influence due to the camera location. The detection probability of cameras located in Sedgwick Forest was about 2.5 times higher than for the cameras in Diamond Hill with no influence of whether near or far from a nest box. Thus, these data do not support our hypothesis that sugar glider detection would be influenced by the presence of nest boxes.

**TABLE 5:** Comparison of detection models from camera‐trapping for the inland sugar glider.

Model	AICc	∆AICc	*W*	*K*
p(2 seasons+ Sedgwick)	947.04	0.00	1.00	4
p(Sedgwick)	959.03	11.99	0.0	3
p(3 locations)	961.31	14.27	0.0	4
p(2 seasons)	984.22	37.18	0.0	3
p(.)	994.50	47.46	0.0	2
p(camera)	996.81	49.77	0.0	3

*Note:* Model parameters as for Table 1. Covariates: 2 seasons, winter contrasted with other seasons; 3 locations; contrast of nest box, far from nest box and Sedgwick (no nest box) sites; Sedgwick, contrast of Sedgwick with Diamond Hill sites; camera, contrast of 2 camera models.

## 4 Discussion

Our study is one of only a few to investigate whether artificial cavities can support a population of non‐flying mammals. This is surprising given a long period of concern about anthropogenic disturbances driving a reduction in the availability of large old trees and the cavities they contain (e.g., Menkhorst 1984; Jackson and Jackson 1986; Lindenmayer, Cunningham, Tanton, and Smith 1990; Newton 1994). Our motivation was to demonstrate that such a study can be conducted at a relatively large spatial scale and that the value offered by the artificial cavities can be evaluated by reference to several basic criteria (see questions below). One feature that distinguishes our study from others is that most other studies have been conducted at a plot or stand scale (e.g., Carey 2002, 13 ha; Williams et al. 2013, 9 ha; Goldingay et al. 2015, ~20 ha; Goldingay 2020a, 9 ha). There are several limitations to our approach (see limitations below), but our findings support the contention that the local populations of our two target species have benefited in a substantive way by the provision of artificial cavities through a large patch of forest.

### 4.1 Were the Artificial Cavities Used by Many Individuals of the Target Species, and Was Use Sustained Over Multiple Years?

Whilst many studies of cavity‐dependent birds have provided findings of many individuals of a target species using artificial cavities, and with use sustained over time (Darling et al. 2004; Berthier et al. 2012; Brazill‐Boast et al. 2013; Saunders et al. 2020), relatively few involving mammals have done so (e.g., Bender 2011; Goldingay et al. 2015; Goldingay 2020a). Typically, the percentage of boxes used by target species has been low in studies of non‐flying mammals (Lindenmayer et al. 2009; Madikiza et al. 2010; Williams et al. 2013; Shuttleworth and Schuchert 2014; Kim et al. 2019). Furthermore, studies have often been of short duration (< 3 years) (e.g., Durant et al. 2009; Best et al. 2022).

We recorded up to 58 adult phascogales and 232 adult inland sugar gliders in nest boxes in a single year. The brush‐tailed phascogale is a solitary species and occupies large home ranges (females > 40 ha; males > 100 ha) (Soderquist 1995; Rhind 2003), so few individuals can be expected, even within a large area of forest. In contrast, sugar gliders live in small groups of 2–7 adults and occupy home ranges of 3–6 ha (Suckling 1984; Quin et al. 1992), so their abundance will be much higher than that of the brush‐tailed phascogale. Therefore, a difference in abundance between species highlights the point that evaluation of cavity use must consider a species' average density and the spatial distribution of the installed cavities.

The mean probability of occupancy per year over our 6 study years was 0.66 (range in naïve values 0.31–0.58) in the phascogale (all individuals) and 0.89 (0.70–0.89) in the sugar glider, demonstrating frequent and sustained use over time. These are very high values compared to other studies on these species and Australian mammals more generally (Table 6). One explanation is that the high values are influenced by the low density of tree cavities (see Goldingay 2025) and particularly those adequate for breeding in our study (Goldingay et al. 2024). The scale of our study (number of sites and boxes) was also greater than in most of these other studies.

**TABLE 6:** The number of nest boxes (B) or sites (S) in studies of Australian mammals describing the percentage of those occupied.

Species	Sites (S)	Boxes (B)	Detected	References
Brush‐tailed phascogale	74	164	31%–58% (S)	This study
	170	170	< 25% (S)	Rhind (2003)
	40	120	8%–23% (S)	Goldingay, Quin, et al. (2020)
	150	450	7%–15% (S)	Lawton et al. (2022)
	31	52	12% (B)	Quin et al. (2021)
Inland sugar glider	74	164	70%–89% (S)	This study
	40	120	65%–98% (S)	Goldingay, Quin, et al. (2020)
	150	450	52%–73% (S)	Lawton et al. (2022)
	30	60	< 5% (S)	Lindenmayer et al. (2016)
	13	48 (CH)	10% (CH)	Best et al. (2022)
	11	150 (CH)	3% (CH)	Honey et al. (2021)
Coastal sugar glider	na	403*	< 34% (B)	Goldingay, Rohweder, and Taylor (2020)
	9	27*	7% (B)**	Miritis et al. (2026)
Savanna glider	99	198	21% (S)	Woolley et al. (2024)
Leadbeater's possum	28	150	11% (B)	Harley (2004)
Common brushtail possum	20	120	9%–10% (B)	Harper et al. (2005)
Eastern ringtail possum	20	120	9%–10% (B)	Harper et al. (2005)
Eastern pygmy‐possum	16	48	0%–69% (S)	Goldingay (2020b)
Feathertail glider	45	135	25% (B)	Goldingay et al. (2007)
Southern greater glider	7	30	100% (B)	Gracanin et al. (2025)
	27	54	63% (S)	Ridley et al. (2025)

*Note:* Detected shows the percentage of sites or boxes occupied. Two studies installed chainsaw hollows (CH) rather than nest boxes.

Abbreviation: na, not available.

* Suitable boxes.

** Animals or their nests.

The early years of our study involved a severe drought (rainfall was 46% of average in 2019), which could be expected to reduce breeding and survival. Our detection probabilities support this hypothesis because variation in abundance can produce variation in detection probability (Royle and Nichols 2003). The detection probability for all phascogales in 2020 and adult females in 2019 and 2020 was 45% of its maximum over the 6 years. Mark‐recapture analysis of this species has revealed declines in abundance with rainfall deficits in box‐ironbark forests (Holland et al. 2012). Detection probabilities in the inland sugar glider in 2018 and 2019 were 66% of its maximum over the 6 years. Elsewhere, the detection probability in the coastal sugar glider showed a marked decline following the 2019 drought year, whereas occupancy did not vary (Goldingay et al. 2025, 2026). Thus, evaluation of the dynamics in the use of artificial cavities needs to consider extrinsic factors.

### 4.2 Were Nest Boxes Used for Breeding?

For artificial cavities to provide population support, the individuals relying on them must also use them to reproduce. We observed a high frequency of breeding in nest boxes by both species in each year of our study. The brush‐tailed phascogale has a semelparous life history strategy (Cuttle 1982) such that only breeding females were present in our October survey each year, though we detected a few instances where females appeared to have lost young. The number of breeding females varied among years (9–29), with the lowest numbers recorded in the main drought year and the year after. In the sugar glider, we detected breeding in at least 68% of glider groups. Best et al. (2022) recorded this species breeding in 25% of frequently occupied chainsaw hollows, whereas Honey et al. (2021) recorded breeding in all glider groups, but only four were detected.

Non‐flying mammals sheltering in nest boxes will readily breed there as we have shown. There is abundant evidence of this for other Australian species: 139 litters in Leadbeater's possums were recorded over 8 years across 150 nest boxes (Harley and Lill 2007); breeding in 65% of 34 nest boxes occupied by feathertail gliders (*Acrobates frontalis*) (Goldingay et al. 2007); 154 breeding events in eastern pygmy‐possums ( *Cercartetus nanus* ) across 120 nest boxes over 3 years (Goldingay and Rueegger 2018); 66 breeding events within nest boxes by six groups of squirrel glider ( *P. norfolcensis* ) over 10 years (Goldingay et al. 2015); breeding in 70% of occupied nest boxes (Gracanin et al. 2025) and at 12% of occupied sites (Ridley et al. 2025) by southern greater gliders ( *Petauroides volans* ). There is also evidence for other mammals. In Britain, Morris et al. (1990) recorded 20 litters of hazel dormouse ( *Muscardinus avellanarius* ) from 71 nest boxes over 2 years. In Italy, Pilastro et al. (1996) recorded 128 breeding events in the fat dormouse ( *Glis glis* ) from 150 nest boxes over 2 years. In North America, Carey (2002) recorded an average of 6 litters per year over 5 years of northern flying squirrels ( *Glaucomys sabrinus* ) and 5 litters per year of Douglas' squirrel ( *Tamiasciurus douglasii* ) within 128 boxes. However, Shuttleworth and Schuchert (2014) documented just three litters in red squirrels within 60 boxes over 9 years.

### 4.3 What Proportion of the Local Population Might Be Supported by the Nest Boxes?

For artificial cavities to provide a management or conservation benefit they should support a large segment of a local population by providing shelter and breeding sites. There is evidence among studies of birds that demonstrate such support (e.g., Arlettaz et al. 2010; Brazill‐Boast et al. 2013; Briskie et al. 2014; Saunders et al. 2020; Berris et al. 2022; Savidge et al. 2022) but few studies of non‐flying mammals have provided evidence (see below). Defining how large a segment should be to be beneficial would require population modelling and is likely to vary among species. On average over 6 years our nest boxes supported 19 female phascogales and 150 sugar gliders which we estimate represented 44%–56% of what the study area could theoretically support if not limited by tree cavity availability. Our camera‐trapping survey suggested the phascogale was limited by tree cavity availability with detection probability 40%–50% lower at sites without nest boxes. This finding was not replicated in the sugar glider with camera detection 2.5 times higher in the Sedgwick Forest with no nest boxes compared to Diamond Hill. The overall low camera detection value in Diamond Hill of sugar gliders (0.06 ± 0.01) compared to that of phascogales (0.35 ± 0.02) is not consistent with the abundance of each species in the nest boxes or the detection probability of sugar gliders in nest boxes (> 0.60). Across our 6‐year study the number of sugar gliders observed was 3.8 times higher than that of phascogales. It is plausible that the camera bait station was too low (1.5 m above ground) to be fully attractive to sugar gliders whereas phascogales go to the ground to cross between trees, so the height was suitable for detection. Our cameras detected red foxes ( *Vulpes vulpes* ) on the ground at all sites in Diamond Hill (three times more than at Sedgwick) which may have posed a greater deterrent to foraging low by gliders but not phascogales.

There is other evidence of artificial cavities providing population support to the inland sugar glider. Two isolated populations were created by the translocation of individuals to small regrowth forest patches (< 150 ha), deficient in tree hollows (Suckling and Goldstraw 1989; Irvine and Bender 1997). These populations, supported by nest boxes, have persisted for at least 30–45 years (Suckling and Goldstraw 1989; Irvine and Bender 1997; Goldingay 2025). An example of short‐term (3 years) population support involves the eastern pygmy‐possum. ‘Large’ artificial cavities were highly preferred by breeding females (Goldingay 2020a). Provisioning of 9‐ha study plots with large cavities increased the odds of a female breeding by 6.5 times and increased the abundance of adult pygmy‐possums overall.

### 4.4 Limitations of This Study

Our study had two key limitations. First, it was conducted in a single 600‐ha forest patch. This scale precluded replication of treatment and control areas. Other studies involving nest boxes with such a design have replicated at the plot or stand scale (see Brady et al. 2000, 13 ha; Carey 2002, 13 ha; Aitken and Martin 2012, 7–20 ha; Ransome and Sullivan 2004, 20 ha; Priol et al. 2014, ~1 ha). In our last year we used camera trapping to compare our experimental forest to a nearby large forest patch without nest boxes. This provided evidence for the brush‐tailed phascogale (but not the sugar glider; see above) suggesting its abundance was greater where nest boxes were provisioned. We chose not to conduct live‐trapping to make this comparison due to the much larger effort required to do this effectively (e.g., Marks and Goldingay 2023) and being aware that live‐trapping of brush‐tailed phascogales can be unreliable (e.g., Holland et al. 2012). The generality of our findings will require additional landscape‐scale studies.

Second, we did not tag any animals within the nest boxes. This was partly to minimise disturbance but also to enable all boxes to be checked within a 2‐day period. Leaving animals untagged meant that we could not investigate patterns of occupation of boxes by individuals or estimate survival over time. Whilst such information could provide valuable insights it does not detract from our objective to evaluate whether nest boxes provided population support. Elsewhere, survival in the squirrel glider was found to increase with greater use of nest boxes when gliders had access to tree cavities and nest boxes (Goldingay 2017). Phascogales have short lifespans (< 18 months; Cuttle 1982; Soderquist 1993b), reducing the number of occasions marked individuals could be encountered and estimates of survival may have had limited value.

### 4.5 Management Implications

There are several important implications from this study. Firstly, our study occurred where there was an absolute shortage of natural tree cavities of sufficient quality required by our target species for breeding (Goldingay et al. 2024). Breeding females of many non‐flying mammals require more space (i.e., greater cavity volume) than non‐breeding individuals (e.g., Goldingay 2020a) to accommodate developing young and a nest that will provide insulation. Female brush‐tailed phascogales may be extreme in this regard by constructing an elaborate enclosed chamber of shredded bark, feathers and other materials (Soderquist 1993a, 1993b; Goldingay, Quin, et al. 2020; Goldingay et al. 2024; Figure 7a). Consequently, artificial cavities may become highly preferred by target species because they provide better quality breeding sites compared to available natural sites (e.g., Arlettaz et al. 2010; Brazill‐Boast et al. 2013; Saunders et al. 2020). Secondly, artificial cavities can support a large segment of a local population of cavity dependent species. We estimated our nest boxes supported at least 44%–56% of the populations of the two species. Such support may be critical for species to have viable local populations. For example, the frequent breeding in our nest boxes should have contributed significantly to the local population because equivalent‐sized natural breeding sites were rare. This benefit may have been particularly critical during the drought period at the start of our study when both species showed evidence of lower abundance. Thirdly, there is legitimate concern about how large a local population might be to be viable. However, our study area is part of a metapopulation for both species, with forest cover in the wider landscape ~5 times that of our study area. The type of cavity augmentation in our study area has been scaled up in the wider landscape with relatively minimal effort (K. Thomas, unpublished data) and therefore will support a much greater number of individuals than reported here. For the inland sugar glider there is evidence that local populations smaller than the present study population (based on forest patch size) and supported by nest boxes have remained extant after 30–45 years (Goldingay 2025). Furthermore, there is genetic evidence for the coastal sugar glider in a landscape with comparable fragmentation to our study landscape showing genetic diversity, and gene flow was maintained despite the fragmentation (Knipler et al. 2023). Further studies like ours on other species of cavity dependent non‐flying mammals will enable greater insight into the value of such artificial cavity interventions.

## Author Contributions

**Ross L. Goldingay:** conceptualization (equal), data curation (equal), formal analysis (lead), investigation (equal), methodology (equal), project administration (lead), supervision (lead), validation (lead), visualization (lead), writing – original draft (lead), writing – review and editing (lead). **Karen J. Thomas:** conceptualization (equal), data curation (equal), investigation (equal), methodology (equal), project administration (equal), resources (lead), supervision (equal), validation (equal), visualization (equal), writing – original draft (supporting), writing – review and editing (supporting). **Darren G. Quin:** conceptualization (supporting), data curation (supporting), investigation (supporting), methodology (supporting), writing – original draft (supporting), writing – review and editing (supporting).

## Funding

The authors have nothing to report.

## Conflicts of Interest

The authors declare no conflicts of interest.

## Data Availability

Data used for analysis in this study are accessible at Dryad https://doi.org/10.5061/dryad.j3tx95xwd.
